# Assessing Risk Acceptability and Tolerability in Italian Tunnels with the Quantum Gu@larp Model

**DOI:** 10.3390/e26010040

**Published:** 2023-12-30

**Authors:** Massimo Guarascio, Emin Alakbarli, Carlota Despabeladera, Vincenzo Cardinale, Azadeh Ghasemichamazkoti, Nima Darabi

**Affiliations:** Faculty of Civil and Industrial Engineering Sapienza, Via Eudossiana 18, 00184 Rome, Italy; massimo.guarascio@uniroma1.it (M.G.); despabeladera.1935920@studenti.uniroma1.it (C.D.); cardinale.1611655@studenti.uniroma1.it (V.C.); ghasemichamazkoti.1955848@studenti.uniroma1.it (A.G.);

**Keywords:** risk indicators, quantum of risk, risk quantum, individual risk, societal risk, expected number of fatalities, scenario, Gu@larp, road tunnel

## Abstract

Road tunnels are associated with numerous risks including traffic accidents and fires, posing threats to individual or group users. Key risk indicators such as Risk Quantum, Individual Risk, Societal Risk, and Expected Number of Fatalities are instrumental in evaluating the level of risk exposure. These indicators empower Rights-Holders and Duty-Holders to report hazards, prevent disasters, and implement timely remedial measures. A crucial indicator, the Scenario Risk Quantum, has its roots in the forensic evaluation of responsibility in a fatal tunnel accident in the UK since 1949. The Quantum of Risk of each design scenario, reasonably selected among rational and practicable possibilities, has both a deterministic and probabilistic character. The Risk Tolerability and Acceptability criteria are modelled according to risk indicators by selecting the parameters according to ethical principles and societal policy. Scenarios are meticulously identified, described, probabilised and assigned probabilities prior to the quantitative risk analysis. These risk indicators are integral to the risk assessment process. This article delves into the understanding of these indicators within the context of Italian road tunnels, employing the Quantum Gu@larp Model to analyse Risk Acceptability and Tolerability.

## 1. Introduction

The evolution of infrastructure development in the 21st century has been marked by an ambitious drive to overcome geographical barriers, leading to an unprecedented rise in the construction of road tunnels worldwide. These subterranean passages present a unique set of challenges, particularly in the field of safety and risk management. From the tunnels of metropolitan cities to the passages carved into the mountains, each tunnel presents various risks, ranging from vehicle accidents to more serious hazards like fire [1], seismic risks [2], and structural failure [3].

It has recorded a few of the most infamous tunnel accidents that have become a critical issue for politicians and for the public, pushing the European Commission to establish the Directive 2004/54/EC that specifically tackles the safety in road tunnels in TERN and aims to guarantee a minimum level of safety to the tunnel users [4]; the major events include the Monte Bianco tunnel (France–Italy) in 1999, the Tauern tunnel (Austria) in 1999 and the St. Gotthard tunnel (Italy–Switzerland) in 2001 [5].

On 24 March 1999, a fire occurred in the middle section of the Monte Bianco tunnel that was initiated by the ignition of an HGV’s lorry, which led to the burning of 34 vehicles including 20 HGVs. It took 39 lives, involving 1 fire-fighter, and left severe structural damage of over 900 m of the tunnel vaults, tunnel equipment, and secondary lining.

On 29 May 1999, just two months after the Monte Bianco fire, a large fire occurred in the Tauern tunnel. The fire was initially due to a front-rear collision of an HGV with passenger cars and an HGV carrying lacquer tins causing a fuel spill on the roadway, which accelerated the development of fire to neighbouring vehicles. This fire incident cost 12 lives and 500 m of material damage, which included road pavements and slabs, tunnel equipment and secondary lining.

On 24 October 2001, a fire accident occurred in the St. Gotthard tunnel due to the collision of two HGVs travelling in opposite directions, causing a fuel spill from one of the HGVs. The fire accident has caused 11 deaths and 8 injuries, and damage to the intermediate ceiling for almost 250 m in the fire zone.

Tunnels represent a unique environment wherein it is feasible to identify maps of exposed people units in a representative manner. Consequently, hazard scenarios can be effectively simulated in thermofluid-dynamics computational environments (Fire Dynamics Simulator), which produce the lethality condition along the time and along the tunnel, in line with traffic flow that has been both empirically and statistically analysed. This specific setting allows for scenarios to be identified in a representative manner, which explains why countries like Italy have considered both Acceptability and Tolerability standard criteria for this specialised environment. In comparison, open-air contexts, such as those governed by the Seveso Directive, have their own set of considerations. The Seveso III Directive (2012/18/EU) [6,7] oversees the control of major-accident hazards involving dangerous substances. Its primary goal is to prevent significant industrial accidents and limit their consequences for human health and the environment. Chemical industries, especially those subject to the Seveso Directive, apply the concept of managing safety concerns arising from the potential releases of hazardous materials. Such releases could escalate into fires, explosions and toxic dispersions [8]. While the ALARP (As Low As Reasonably Practicable) model for underground systems and the Seveso Directive for chemical/high-hazard industries each have their distinct domains of application, their principles overlap in the broader context of risk management. This is especially true in scenarios involving tunnels, where the transportation of dangerous goods poses significant safety concerns. Within these tunnels, individuals find themselves trapped in confined spaces, potentially exposed to toxic smoke diffusion, further amplifying the importance of integrated risk management across both domains.

The intrinsic characteristics of tunnels—limited lighting, insufficient ventilation, and confined spatial parameters—serve as catalysts for exacerbating the hazards associated with fire outbreaks [1,9]. Upon the initiation of a fire, the swift proliferation of toxic smoke within the tunnel’s confines poses formidable challenges to evacuation and rescue operations, thereby endangering the safety of occupants [10,11]. Such fire accidents transcend the immediate peril, manifesting in substantial economic repercussions and engendering profound societal impacts. This alarming reality has propelled many European countries to adopt a pragmatic approach where quantitative risk acceptance criteria are employed for road tunnels, a practice galvanised by the critical fire events in Mont Blanc, Tauern, and St. Gotthard tunnels that spotlighted the exigency of enhanced fire safety measures within tunnels [1,5].

- In Slovenia, a study aimed to determine the risk acceptability criteria and risk analysis of two road tunnels where a temporary traffic regime was introduced due to reconstruction. The study acknowledged that numerous factors influence risk, although the details on the specific criteria were not fully elaborated [12];
- In Austria, the guideline “RVS 09.03.11” [13,14] introduces the Austrian Tunnel Risk Analysis Model “TuRisMo”, which defines how to assess the risk for tunnel users. It also stipulates that the specific risk involved in the transport of Dangerous Goods (DG) through road tunnels should be assessed in a separate process [15];
- In Slovakia, a study was conducted accordance with the technical specification TP 02/2011, titled “Risk analysis for Slovak road tunnels”. It focused on utilising tunnel simulation tools for a variety of operational scenarios, particularly emergency situations. The study also quantified the expected value of risk in road tunnels, expressed as the annual expected number of fatal casualties [16];
- In the Netherlands, Acceptability criteria have been adopted for both individual risk and societal risk. For example, the necessity to maintain safety distances between LPG activities and vulnerable objects like houses is emphasised, with the safety distances based on Quantitative Risk Assessment (QRA) and Risk Tolerability criteria [17];
- In the UK, in proposed immersed road tunnels, the Risk Acceptability criteria were developed based on an analysis of hazard categories. The Dual Risk Acceptability criteria focused on the aggregate risk per annum to any regular user and societal risk criteria, represented by an F-N curve, relying on ALARP (where Risk Quantum Scenario are defined) dynamics to mitigate risk [18,19,20];
- In Italy, a model based on the ALARP principle is employed for tunnel risk-based design, particularly in scenarios of fire accidents. The ALARP Risk Acceptability and Tolerability criteria require verification to ensure that a minimum-sufficient level of safety is guaranteed within the tunnels [19,20].

Many European countries adopt quantitative criteria for evaluating the acceptable risk in road tunnels, often expressed by FN-criteria and IR-values [21], although this approach can be challenging to adopt in practice due to the extensive analyses and documentation required for different types of tunnels. Different types of risks are addressed through a risk-based approach, including societal risk, individual risk, loss of property, and environmental damage. A significant number of European countries adhere to the European Agreement concerning the International Carriage of Dangerous Goods by Road (ADR) [22], which includes specific arrangements for tunnels. EU countries are bound by Directive 2004/54/CE [4], which prescribes a minimum level of safety arrangements for road tunnels longer than 500 m as part of the trans-European road network. However, the EU does not provide risk acceptance criteria for tunnels, leaving it up to individual nations to define these criteria as previously mentioned (i.e., the approaches taken in Slovenia, Austria, Slovakia, the Netherlands, the UK, and Italy). There is not a universally accepted set of risk criteria for road tunnels. The calculated risks are compared to well-defined risk tolerability criteria, and the approach to risk analysis and acceptance varies between major European tunnel projects. In light of addressing the concerns surrounding the variability and complexity in evaluating acceptable risk in road tunnels, in the subsequent Section 2, Section 3 and Section 4, we provide a detailed explanation of the GU@larp mathematical model, elaborating on its role in establishing precise risk acceptance/tolerable criteria and immediate quantification of the four critical risk indicators—Risk Quantum, Individual Risk, Societal Risk and Expected Number of Fatalities.

Italy, with its rich tapestry of landscapes, from the rugged Alps in the north to the rolling hills of Tuscany, boasts an intricate network of road tunnels. These tunnels, vital for connectivity and commerce, are emblematic of Italy’s commitment to infrastructural excellence. However, their safety remains an area of intense scrutiny and research. Historical accidents, some leading to tragic outcomes, have accentuated the need for a more profound, data-driven understanding of tunnel-related risks. The lessons from past accidents serve as a sombre reminder of the consequences of oversight and the indispensable nature of pre-emptive risk assessment [23].

In the broader academic and professional discourse on civil engineering, the topic of tunnel safety has garnered significant attention [19,20,24,25,26,27]. The multifaceted nature of risks, influenced by factors such as tunnel design, traffic density, geographical location, and maintenance protocols, necessitates a holistic approach to risk assessment. This approach must seamlessly integrate empirical observations, advanced probabilistic models, and scenario-based analyses to predict and mitigate potential hazards effectively [28,29,30,31].

Risk assessment requires a clear understanding and application of conceptual probability [8,32,33,34,35], and the indicators for this are detailed in our paper. Our research rigorously addresses the concept of risk. We have integrated innovative techniques to distinctly identify each risk variable, ensuring that Hazard and Risk Scenarios are meticulously identified and described. Our primary contribution is in demonstrating how to use both the concept of probability and actual probability numbers in tandem. The key innovation lies in differentiating two main components: the Probability value and the description of the potential event namely Hazard and Risk Scenario. This distinction allows us to quantify the risk associated with each event or scenario occurrences. By aggregating these risk quantifications, we provide a comprehensive characterisation of the entire event space or more properly in our domain “Scenario space”. The estimated probabilities of the above group of scenarios have to be coupled with a corresponding mutually exclusive group of complex events described by the scenarios themselves.

The PIARC review documents (2008 and 2022) emphasise that an acceptable level of safety is crucial for ensuring the performance level of a given protection system in road tunnels. It focuses on enhancing road tunnel resilience by addressing concerns related to safety and the availability of protection systems [24,36].

The literature presents various models and approaches to define the limits of Risk Acceptability or Risk Tolerability. One widely recognised approach is the ALARP approach, which is seen as vital for managing risks in various sectors, including road tunnels. One of the reviews carried out between 2013 and 2015 highlights those publications regarding the risk impacts of their level of Acceptability or Tolerability [37].

This study embarks on a comprehensive exploration of risk assessment methodologies tailored for Italian tunnels. By introducing innovative risk indicators and leveraging cutting-edge modelling techniques, the research aims to provide a blueprint for enhancing tunnel safety not just in Italy but potentially for underground networks worldwide. As urbanisation intensifies and the demand for efficient transportation grows, the insights from this research will be pivotal in shaping safer, more resilient underground infrastructures for the future.

The research group of this paper is now extending the investigation according to perspectives and directions:

- Risk-based design considering underground facilities and gatherings exposed to multiple hazards (seismic-structural-fire and technological failure).
- Risk assessment and validation in real-time using continuous video recorded data and the Supervisory Control and Data Acquisition (SCADA) in progress in tunnel systems in central Italy and underground facilities worldwide.

## 2. Risk Indicators and Equations

In the domain of this paper, road tunnel safety, risk is the fusion and synthesis of two fundamental but complementary concepts: the unexpected side and the undesirable side of possible hazardous occurrences. In particular, an appropriate number of scenarios must be identified, which is both necessary and sufficient for risk analysis purposes. Scenarios are not observable entities. In risk-based safety design practice, scenarios are identified according to basic hypotheses or conjectures. Risk indicators are important forerunners of undesirable or unexpected tunnel events that may have a detrimental impact on users, governments and duty-holders.

We define here the equations of the four basic risk indicators where the first, the Scenario Risk Quantum, allows us to obtain the others [20].

- The Risk Quantum *Q*(*N_i_*) is the product of the probability of each scenario *p*(*S_i_*) times the number of fatalities in the same scenario *N*(*S_i_*) = *N_i_*.

(1) $$Q(N_{i})=p(S_{i})\;\times N(S_{i}),$$

- Exceedance probability *F*(*N_i_*), also named Societal Risk, is the Probability of the Union of Scenarios whose number of fatalities (*N*) exceeds a given threshold $\bar{N_{i}}$ [38,39,40,41,42].

(2) $$F(N_{i})=\sum_{i}p(N\;\geq \;\bar{N_{i}}),$$

- Individual Risk *q*(*Ni/Exp*) is the probability that an individual will lose their life in a tunnel accident (scenario) if exposed once a year corresponding to the Risk Quantum of the above scenario divided by exposed people units in the considered scenario itself.

(3) $$q\left(\frac{N_{i}}{Nexposed}\right)=\frac{\mathrm{Risk}\;\mathrm{Quantum}\;Q\left(N_{i}\right)}{\mathrm{Exposed}\;\mathrm{units}\;\mathrm{in}\;S_{i}}=\frac{p\left(S_{i}\right)\cdot N\left(S_{i}\right)}{\mathrm{Exposed}\;\mathrm{units}\;\mathrm{in}\;S_{i}}=\frac{F\left(N=1\right)-F(N=2)}{\mathrm{Exposed}\;\mathrm{units}\;\mathrm{in}\;S_{i}},$$

- Expected Number of Fatalities *E*(*N*) in the Union of all the Design Scenarios corresponding to the sum of the Risk Quantum of the above union.

(4) $$E(N)=\sum Q(N_{i}),$$

The four definitions and equations described above therefore provide the four fundamental numerical risk indicators for all the purposes of risk analyses.

## 3. ALARP Concept

Among the various criteria used to evaluate the risk, the ALARP concept is in agreement with a lot of good properties for the risk management in many sectors of civil and industrial engineering systems [27]. The ALARP principle essentially “involves weighing a risk against the trouble, time and money needed to control it”. A decision as to whether the use of risk reduction strategies is grossly disproportionate to the benefits achieved by the risk reduction must be made. The concept is natural and symmetrical between duty holders and rights holders.

**Figure 1:**
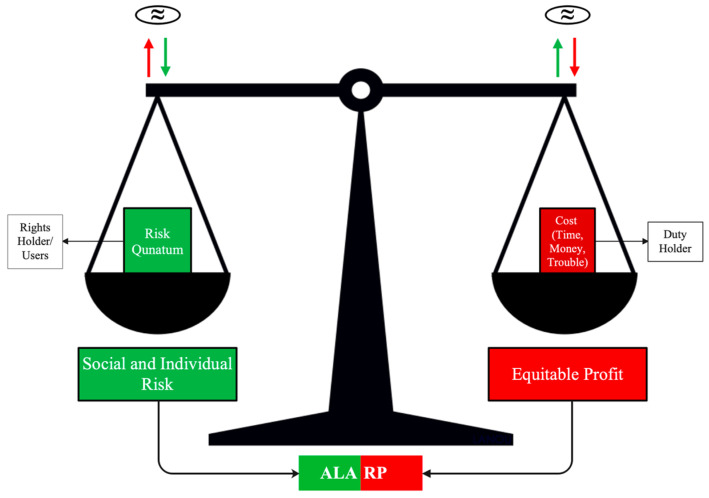
Schematic representation of the ALARP concept when considering the risk and cost of the risk reduction.

A schematic representation of Lord Asquith’s judgment [43], a case of mining mortal accident in a UK Court judgment (Edwards vs. National Coal Board 1949), is presented in Figure 1. In this judgment, Lord Asquith introduced the term “Quantum of Risk” to emphasise the need for a specific, quantifiable measure of risk. This measurable degree of risk is crucial for making a computation in which the risk is weighed against the sacrifice involved in averting it. The use of “Quantum” underscores the necessity of an objective assessment, ensuring that decisions are made based on concrete evaluations rather than vague perceptions. This approach ensures that risks are managed to achieve the ALARP conditions [44].

Risk is an abstract concept involving the deterministic term measuring the number (*N*) of fatalities on one scale and probabilities of occurrence of scenarios involving the above number (*N*) of fatalities on another scale.

The concept of equilibrium between the strategies of the rights holders (as low as) and duty holders (reasonably practicable) might be in agreement with the Game Theory basic concept as the Pareto-type Optimality and Nash Equilibrium [45].

The recommended Disaster Risk Reduction (DRR) strategies in the case of ALARP allow for the implementation of such measures as long as they are reasonably proportionate to the Risk Reduction efforts and not grossly disproportionate. To describe the Lord Asquith equation in probabilistic quantitative terms, we could state the following:

ALARP = min(Risk|Equitable Cost) = min(Cost|Acceptable Risk),(5)

Here, the symmetrical probabilistic equation below represents the “Bayes’ theorem”:

P(R|EC) × P(EC) = P(C|AR) × P(AR),(6)

Consequently, we obtained the “Bayes’ theorem” condition:

P(EC ∩ AR) = P(R|EC) × P(EC) = P(C|AR) × P(AR),(7)

It means that in order to achieve ALARP conditions, an optimisation in the common domain of Equitable Cost and Acceptable Risk should be carried out.

## 4. Risk Functions: Acceptability and Tolerability Criteria

Accidental uncontrollable events aside, unexpected risk arises from a lack of risk-based design knowledge or from an implementation error that could cause an unexpected event. The above leads to the need to model the probabilistic predictability of consequences, including the estimation of the probability of “complex events”, such as scenarios. When the risk cannot be predicted or avoided, people cannot be protected and the events cannot be prevented.

The limits of Acceptability and Tolerability are assumed according to the decisions of the authorities of the countries. The implementation of both criteria aims to recognise the equilibrium between undesirable consequences and acceptation of a disutility. As a result, some people could be exposed to risk, and one must assume a limit for the risk that works for the overall advantage of society as a whole.

A risk exists if or when there are hazards that may occur at a given moment in time and at a specific site. The risk may appear as an abstract concept because the effects are not observable in details: what is not observable can be adequately modelled and simulated according to an appropriate number of representative scenarios, as we discussed above.

The level of risk is then measured quantitatively by the probability of the potential number of human losses (fatalities (*N*)) given a specific Hazard Scenario [26]. To approach risk analysis in a quantitative manner, it is necessary to estimate the probability of hazardous events, i.e., through their observed hazard rate, and related hazard and exposure scenarios.

In tunnel case studies, the identification and selection of necessary and sufficient number of scenarios are based on the combinatorial analysis of several “key major factors”: tunnel length, traffic volume characterisation (AADT, traffic flow, traffic density, average speed, variance among vehicle speed and others), percentage of vehicles with dangerous goods in traffic volume, performance and availability of protection system and others. Combinatorial analysis of the above factors is carried on through the tree diagrams tool and methodology [19,20,24,25].

In identifying scenarios, it is necessary to identify and select a limited number of scenarios that can appropriately represent all possible scenarios that can represent reality. Therefore, scenarios must be characterised by logical factors that have a significant influence on the probability of occurrence and possible consequences, or the factors that affect the Risk Quantum of a given scenario. Most importantly, these logical factors must allow the model to identify, describe and probabilise the scenarios. Given that the fixed characteristics of the tunnel such as geometry (shape, slope, etc.) are given, the model established in this paper also considered a set of “key major factors” including fire rate and fire design, temporal and seasonal characteristics of the traffic, location of the fire and the people inside the tunnel, and the availability of performances of the protection systems [20].

The accident rate related to traffic and eventually the accidents in specific tunnels or roads can be assessed by considering the number of kilometres traveled by vehicles and then transformed in probabilities. The fire rate is also related to the kilometres traveled but could be correlated with the traffic accident. The above-introduced logic takes into consideration as major parameters the tunnel’s length and traffic density [4,46,47]. In the Italian Legislative Decree No. 264/2006 and the Directive 2004/54/EC, the level of safety is guaranteed through mandatory safety and protection requirements that are identified according to the length and volume and composition of traffic; the spirit of the directive is based on the logic connection between the tunnel length and traffic volume with the fire accident probability of occurrence conditioned by the above parameters.

Additionally, accident and the fire rate data have been gathered from tunnel fire statistics across 12 global countries, as reported by the PIARC [48] in studies on accidents in road tunnels. According to the report, most of the ordinary accidents in tunnels are recorded at the entrance. For the scenario identification, only the interior zone and the exit area of the tunnel were considered.

In situations involving fire outbreaks in a unidirectional tunnel, vehicles positioned downstream of the accident site usually remain unaffected, as they possess the advantage of promptly exiting the tunnel. In contrast, vehicles upstream face an increased risk, chiefly due to the potential obstructions originating from the accident. If the fire ignites near the entrance of a unidirectional tunnel, it is noteworthy that a limited number of vehicles will be directly endangered. Moreover, accidents occurring in this specific zone have an incredibly low, nearly insignificant probability of resulting in injuries or fatalities [48]. Individuals close to the entrance can conveniently evacuate on foot, and upon reaching the exterior, they are largely shielded from the perils of smoke. Hence, despite having a high probability of an accident occurring in the entrance area, the consequences are of less significance to the overall risk due to the low probability of resulting injuries or fatalities.

On the other hand, bidirectional tunnels manifest a distinct set of challenges. They inherently harbour a more pronounced risk for accidents, fires included, in comparison to their unidirectional counterparts. Particularly, tunnels built for one-way traffic but operate bidirectionally can foster hazardous conditions, amplified by the elevated potential of severe head-on collisions. When a fire breaks out in such settings, the ramifications are compounded by factors like rising temperatures, increased smoke density, diminished visibility, and the prevalence of toxic elements [49]. This is especially true for those trying to escape downstream from the fire’s epicentre. Vehicles downstream of an accident in either direction usually face minimal hindrances when attempting to exit. However, vehicles upstream, especially in the lane of the accident, encounter heightened risks. Similarly, vehicles traveling in the opposite lane, even if their exit path appears unobstructed, can be exposed to challenges presented by dense smoke, compromised visibility, and potential disarray among drivers. Like unidirectional tunnels, occupants near the entrance in bidirectional tunnels can also evacuate on foot relatively easily. Yet, fires near the entrance pose heightened risks, particularly with smoke impacting vehicles in the opposite lane.

The temporal and spatial characteristic of the accident identifies the initiating events, independent from the influence of the availability and unavailability of performance of the protection systems on the hazard flow. The probability of initiating events is the probability of occurring an accident in a given location inside the tunnel on a specific day and time. Moreover, if the traffic flow is higher in the day than at night, then the number of people that will be present in the tunnel during the day will also be higher. Accordingly, in the event of an accident resulting in fire with a specific power, the number of exposed units and the number of fatalities might also be higher during the day than at night.

Since risk is a function of both the probability of occurrence and the consequence in terms of fatalities, it is possible for scenarios to arise that are highly unlikely to occur, regardless of their consequences, as well as for scenarios to occur that have negligible consequences, irrespective of their probability. This is because both the cases mentioned would give a value of Risk Quantum that is irrelevant to the overall risk of the system and Expected Number of Fatalities.

**Figure 2:**
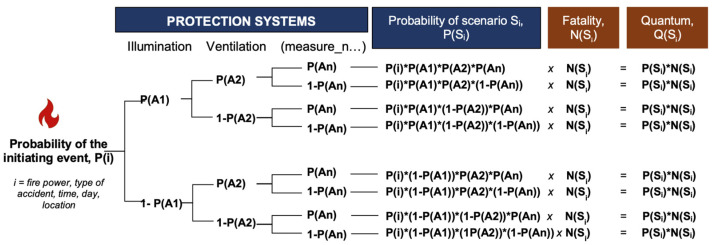
Scenario dissection through tree diagram methodology.

Quantitative characterisation of any single scenario is described in each branch of a tree diagram, as shown in Figure 2. The mortality rate assessment associated with each scenario is estimated through computational simulators (e.g., FDS, Evac, etc.), including deterministic tools that reproduce virtual scenario conditions that can be validated and verified.

The scenario tree diagrams of the various initiating events are the computational tools that allow us to probabilise the scenarios to measure the Risk Quantum of each simple scenario by considering the impact of the performance of the protective system. The tree diagram tool ensures the condition that all the scenarios can be assumed as a “complete group of mutually exclusive complex events”; the probability of the union of all scenarios is equal to the probability of the initiating hazard event, which is a multiplicative numerical factor of the tree diagram. In this context, the branches of a tree diagram represent these distinct scenarios, defining the entire probability space from both probabilistic and mathematical perspectives. For the selected identified scenarios, combinations of key major factors must be necessary and sufficient to represent all the scenarios that can potentially occur. Each factor is carefully probabilised and appropriately weighted to ensure an accurate estimate of probability. The probability of each scenario being given by the corresponding simple tree branch is the product of the probability of the initiating event times along the specific branches. Finally, the number of fatalities in each scenario is estimated by simulation using as input data the scenario characteristics and at the conditions previously identified. The Scenario Risk Quantum is then the result of the probability of the scenario multiplied by the corresponding Number of Fatalities. Additionally, the scenarios are coded in order to facilitate the ranking of their probabilities and corresponding Risk Quantum. Sensitivity analysis can be easily conducted by slightly varying the values of the key major factors and observing the impacts on the Risk Quantum. In Italy, the process of risk analysis is legally mandated by Italian Legislative Decree No. 264/2006, which requires an annual assessment in each tunnel. The availability of specific probability values and corresponding Risk Quantum for each scenario enables the immediate use of data for any kind of validation and verification.

The Expected Number of Fatalities of the overall scenarios can be then derived by the combination of the probabilities corresponding to the tree diagram branches and the corresponding deterministic values of the number of fatalities (*N_i_*) provided by the simulator. The number of fatalities in each scenario must be integer values *N*(*S_i_*), which can be coupled with the probabilities of the corresponding scenarios (*S_i_*). The probabilities of the single (*S_i_*) are indicated in the Gu@larp model as g(*N_i_*(*S_i_*)) or shortly g(*N_i_*). The corresponding exceedance probability is G(*N_i_*) = Gu/*N_i_*, where Gu is equal to G(*N_i_*) = 1. This function is a real staircase-type function, monotonically decreasing, where the variable (*N*) assumes only the integer values. The related exceedance probabilities are defined as $G\left(N_{i}\right)=\sum_{i}g(N\geq N_{i})$. The related probability of a scenario (*S_i_*) can be described as g(*N_i_*) = G(*N_i_*) − G(*N_i_* + 1).

The parent functions in the continuous number domain [19] are the equations of the equilateral hyperbola functions, namely $G\left(x\right)=\mathrm{Gu}\cdot x^{-1}$, where Gu plays the role of parameter. The corresponding density function is:

(8) $$\left\{\begin{array}{c} g\left(x\right)=Gu\cdot x^{-1}\;\;\;\;\;\;\;\;\;\;\;\mathrm{where}\;x\geq 1\\ g\left(x=0\right)=1-\mathrm{Gu}\;\;\;(\mathrm{Dirac}\partial \mathrm{type})\\ g\left(x\right)=0\;\;\;\;\;\;\;\;\;\;\;\;\;\;\;\;\;\;\;\;\;\;\;\;\;\;\mathrm{elsewhere} \end{array}\right.,$$

In practice for representative descriptions of reality, the density function is truncated at an appropriate value x = x_max_. The above assumption does render the expected value E(x) a limited one. To achieve this result, an acceptable approximation is to locate a heaviside step function in the point x = x_max_ equal to $G\left(x_{\max}\right)=\mathrm{Gu}/x_{\max}$. Consequently, the finite expected value is E(x) = Gu(Logx_max_ + 1).

**Figure 3:**
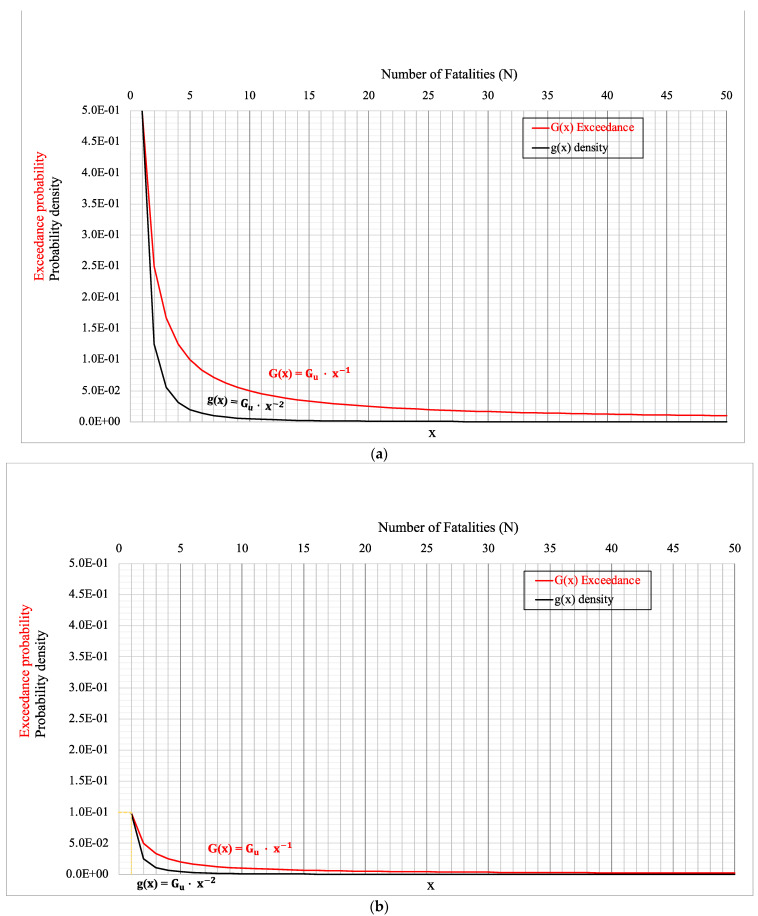

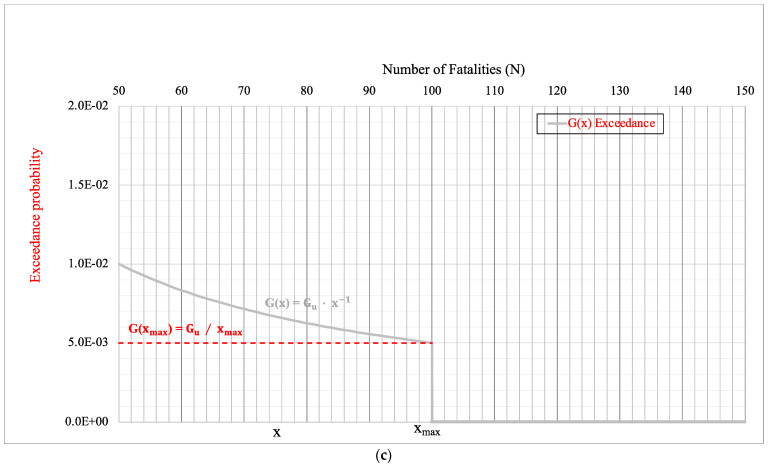
Exceedance probability model assuming: (**a**) Gu = 0.5; (**b**) Gu = 0.1; (**c**) Gu = 0.5 and G(x_max_) = Gu/x_max_ for x ≥ x_max_ values. The red dotted line emphasises the coordinates of the truncation point, which is important for practical applications.

Figure 3 below presents three examples of exceedance probability models.

**Figure 4:**
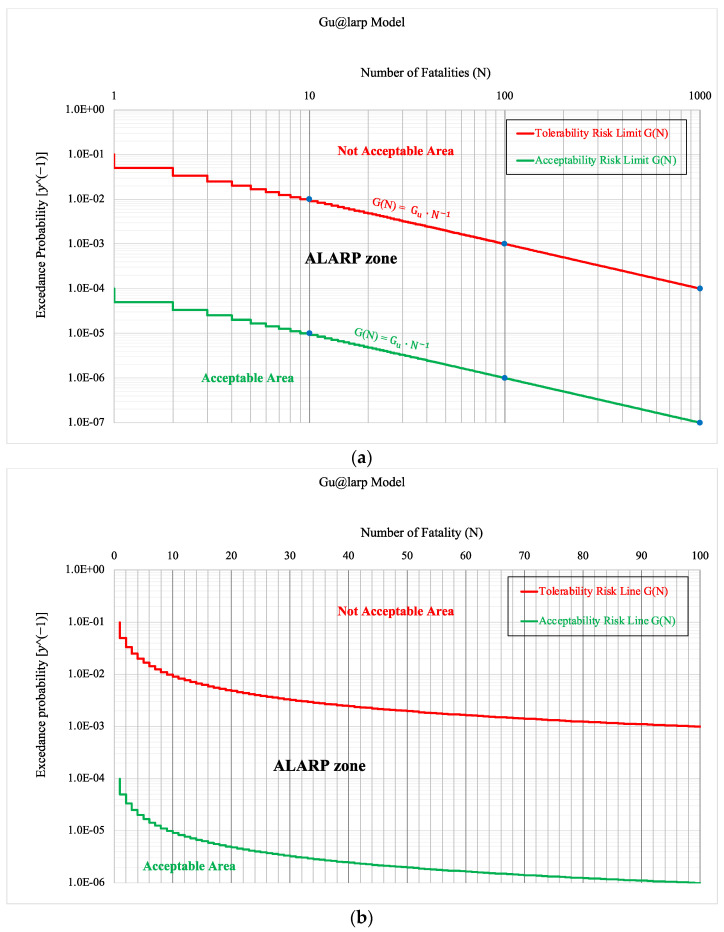
Gu@larp (Italian 264 Decree (2006)) models on logarithmic (**a**); and arithmetic (**b**) scales.

The numerical models of the scenario probability as g(*N*) functions and related exceedance probability G(*N*) are usually represented in a diagram on a logarithmic scale where on the horizontal axis, there are integer values of the number of fatalities (*N*) of the scenarios, and on the vertical axis, there is the probability of exceedance of the integer threshold values (Figure 4a).

The above probability of exceedance is nothing other than the probability of the union of the scenarios with the number of fatalities greater than the threshold value. The relation between the number of fatalities and exceedance probability on an arithmetic scale is also shown in Figure 4b.

The risk function has to be a staircase type in order to describe and calculate properly the Risk Quantum of the single scenarios, defined below for the purpose of a serious and accurate design objective. Once all of the above quantities are estimated, risk indicators could be immediately obtained for all possible hazards and hazardous events on one hand, and traffic, protection systems and fatality scenarios on the other hand.

Here, we present our approach named as the Gu@larp model related to the ALARP principle adopted in the Italian Legislative Decree No. 264/2006 [47] in agreement with the Directive 2004/54/EC [4]. Here, in Table 1, Table 2 and Table 3, there are some examples of numerical values characterising the Tolerability and Acceptability Limit curves adopted in the Italian law [47].

**Figure 5:**
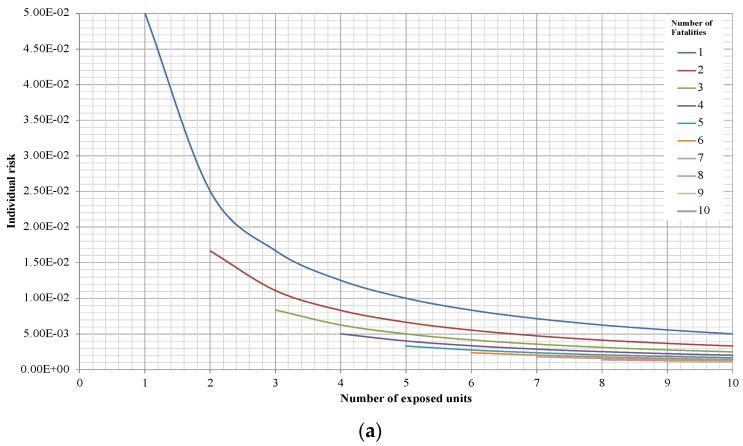

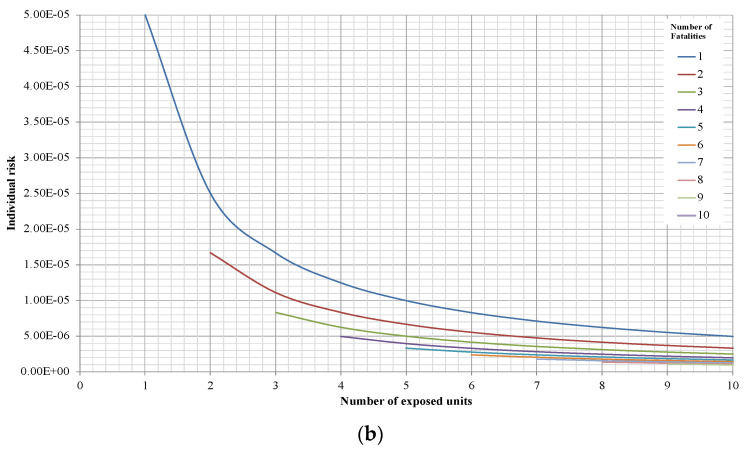
Behaviour of the Individual Risk with respect to number of exposed units, on an arithmetic scale for different values of Gu and with the number of fatalities as parameters in coloured lines: (**a**) Gu = 10^−1^; and (**b**) Gu = 10^−4^.

Figure 5a,b consider the number of fatality intervals from 0 to 10 with reference points given in Table 1. The Individual Risk is taken into account, and its behaviour is observable when considering the number of exposed people, parameterised by the number of fatalities, represented in different coloured lines. The Individual Risks show hyperbolic-type behaviour vs. exposed units, where the highest value corresponds to the minimum number of exposure units, which decreases monotonically as the number of exposed units increases.

**Figure 6:**
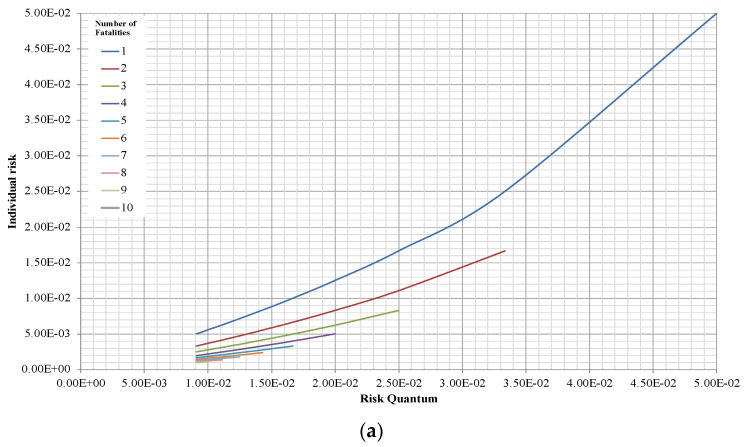

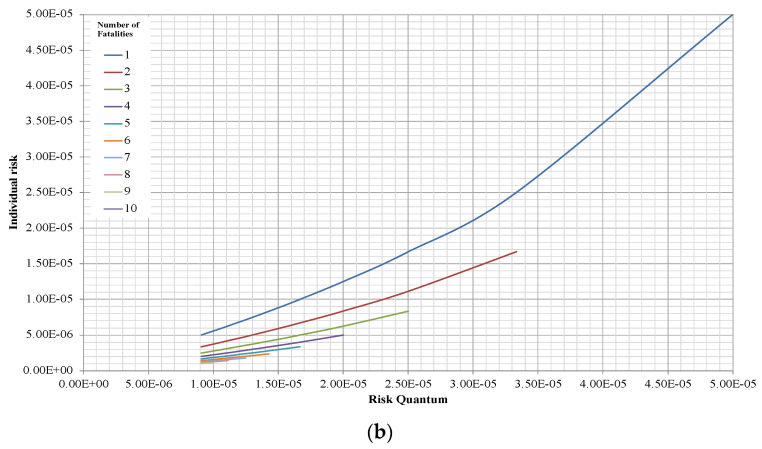
Pattern of the Individual Risk versus the Risk Quantum, on an arithmetic scale. Different numbers of fatalities are represented by different coloured lines: (**a**) Gu = 10^−1^; and (**b**) Gu = 10^−4^.

In Figure 6, we can appreciate how much, for a given value of the Risk Quantum in a scenario, the number of fatalities can range in a wide interval, increasing as the value of the Risk Quantum itself increases and the corresponding Individual Risk will vary accordingly.

### Representation and Acceptance of Risk for the Users in Tunnel Safety Design

For an in-depth analysis of the risk scenarios, one can use the exceedance probability design scenario curve *F*(*N*(*S_i_*)) vs. (*N*) critically compared with the corresponding ones of the Acceptability and Tolerability curves G(*N*(*S_i_*)) vs. (*N*) and *F*(*N*(*S_i_*)) vs. (*N*).

These curves are determined considering the number of people involved in the accident as exposed units and the mortality rate ((*N*) Fatalities) as a consequence of their exposure. The regulation and management of high engineering systems usually require the use of these diagrams. They can be incorporated into a land-use planning policy or in the risk assessment of future installations.

**Figure 7:**
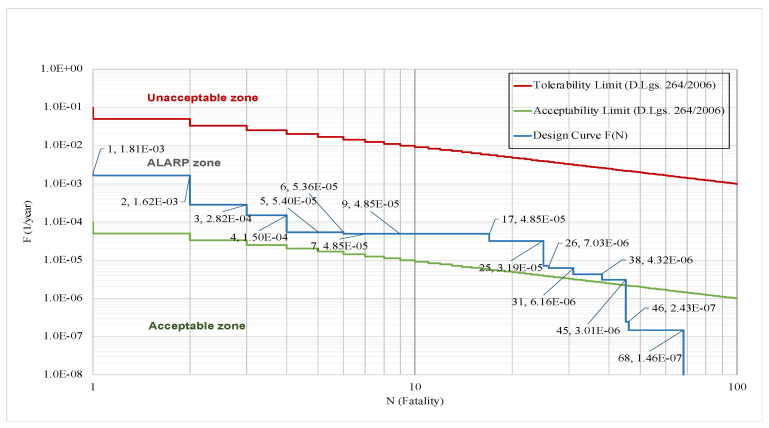
ALARP, Tolerability and Acceptability Limits for the road tunnels in Italy and an example of the exceedance probability curve of a tunnel design (Gu@larp (Italian 264 Decree (2006)) model).

Figure 7 presents the exceedance probability curves of the Tolerability and Acceptability Limit criteria (based on the Italian Decree 264/2006) distributed according to the Gu@larp model. As defined above, the exceedance probability G(*N_i_*) corresponding to a given threshold *N_i_* is the Probability of the Union of the Scenarios with the number of fatalities ≥ *N_i_*, and in which the corresponding reference values of the Tolerability and Acceptability curves at *N* = 1 are Gu = 10^−1^, and Gu = 10^−4^, respectively (Table 1). Also, it can be noted that the exceedance probabilities of the two curves are monotonically decreasing in a way that the probabilities are also decreasing at a given rate, which also decreases as N increases. Using the Tolerability/Acceptability Limit criteria [19], the exceedance probability curve resulting from the design of a specific tunnel can then be evaluated if it is compliant with the said criteria or not, as shown in the above figure.

**Figure 8:**
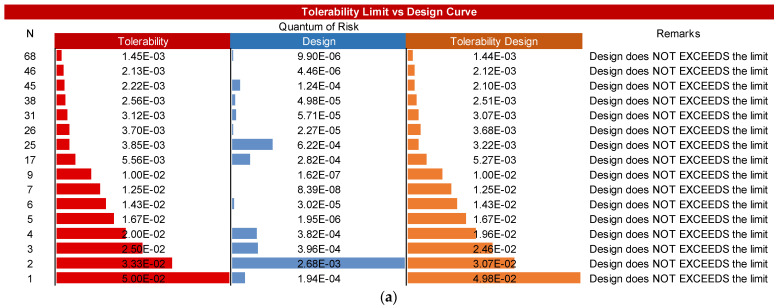

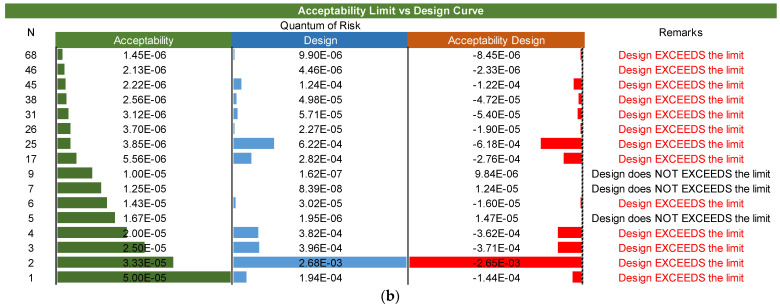
Ranking of the Risk Quantum of scenarios for the tolerable limit vs. design and their difference accordingly number of fatalities: (**a**) Tolerability limit curve; and (**b**) Acceptability limit curve.

Nonetheless, to embody the ALARP spirit in the design phase of a tunnel, the visual comparison of the exceedance probability curves of the limit criteria and of the design is insufficient to conclude the “acceptability” of the risk. Instead, it is also important to compare the quantum of risk *Q*(*N_i_*) of the limit scenario (accepted or tolerated) compared with a design scenario with the same number of fatalities to check the “acceptability” of the scenarios according to their Risk Quantum and doing so to identify the scenario/s that exceed the limit criteria. To better explain the importance of Risk Quantum in the verification of “acceptability”, we offer an example coherent with Figure 7 and described in Figure 8. We can notice that although the design curve shows that the scenarios with fatality numbers *N* = 46 and *N* = 68 are below the Acceptability Limit curve, their quantum values demonstrate where they exceed their corresponding Risk Quantum in the Acceptability Limit. On the other hand, the design curve shows that the scenarios with fatality numbers *N* = 5, *N* = 7 and *N* = 9 are above the Acceptability Limit curve, while their Risk Quantum does not exceed the limit. Hence, it can be concluded that the scenarios with fatality numbers of 1, 2, 3, 4, 6, 17, 25, 26, 31, 38, 45, 46 and 68 are the scenarios that did not pass the Acceptability Limit.

The concept of the Risk Quantum allows us to rank the scenarios, and then quantitatively appreciate the comparative contribution of every single scenario to the overall Expected Number of Fatalities. It should be noted that:

G(*N* = 0) = 1, (9)

G(*N* = 1) = Gu, (10)

And therefore:

g(*N* = 0) = 1 − Gu, (11)

Further:

G(*N* = 1) − G(*N* = 2) = g(*N* = 1) = *Q*(*N* = 1) = *q*(*N* = 1), (12)

**Figure 9:**
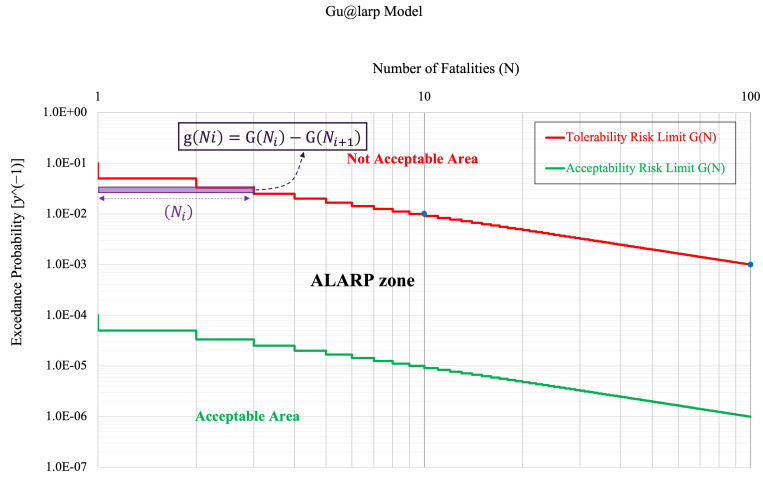

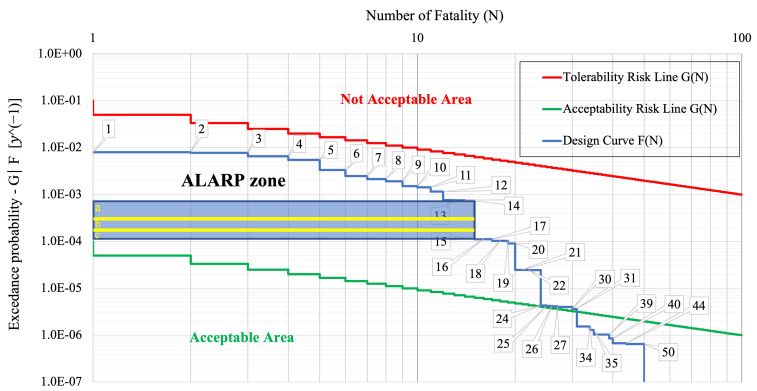
(**a**) Geometrical description of the probability of a scenario (*S_i_*) with *N*(*S_i_*) fatalities. *Q*(*N_i_*) is the corresponding Risk Quantum. The rise of the stair is the Probability of Scenario (g(*N_i_*(*S_i_*))). The area of strip g(*N_i_*) × *N_i_* is the above Risk Quantum; (**b**) example of three design scenarios (divided by yellow lines and labeled as a, b, and c, respectively) with the same number of fatalities corresponding to *N*(*S_i_*) = 14 fatalities.

Figure 9a,b more effectively show the concept of the Risk Quantum of Scenarios in G(*N_i_*) vs. (*N_i_*) and *F*(*N_i_*) vs. (*N_i_*) curves. The above curve has the character (acceptability, tolerability and design scenarios’ exceedance probability) of staircase-type curves. As can be noticed in those curves, the higher the number of fatalities, the lower the exceedance probability will be. This means that the proposed integer function is monotonically decreasing. This conveys that although Ni is increasing, the probability mass density defined as: g(*N_i_*) = [G(*N_i_*) − G(*N_i_* + 1)], which is decreasing at a higher rate than *N_i_*; this causes the Risk Quantum of Scenarios, calculated as: [G(*N_i_*) − G(*N_i_* + 1)] × *N_i_*, to have a declining pattern. It can be seen that the number of fatalities in any scenario of risk can be identified and appreciated as the geometric stair length in purple, whereas the height of rise is the probability of the scenario itself.

The horizontal axis shows the number of fatalities, N, and the vertical axis represents the exceedance probability, G(*N*). The rise of each stair is the measure of the corresponding scenario probability.

**Figure 10:**
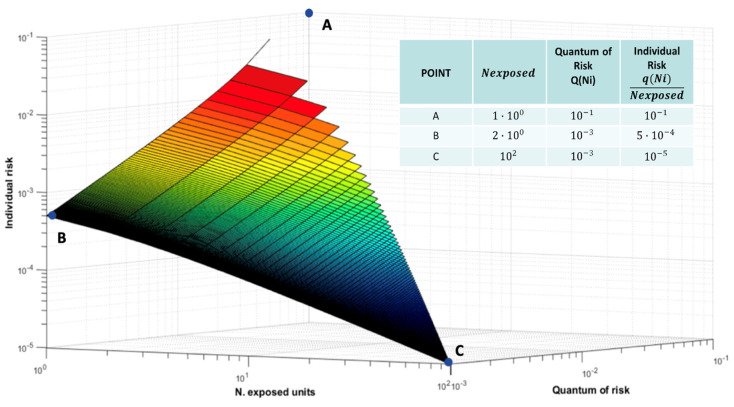
Three-dimensional view of *Q(N_i_)*, *q(N_i_/Nexposed)*, and *Nexposed* on a logarithmic scale, (Gu = 10^−1^).

In Figure 10, we see a 3D view of the relationship, in a given Gu@larp model for the assumption of Gu = 10^−1^, between the Risk Quantum *Q*(*N_i_*)**, the Individual Risk *q*(*N_i_*/*Nexposed*)** and the Number of Exposed units *Nexposed*. For a scenario with a given Risk Quantum, the corresponding Individual Risk shows a relationship inversely proportional to the number of exposed units.

## 5. Conclusions

The environment of risk now encompasses not only traditional scenarios but also introduces the Risk Quantum for each scenario and group of scenarios. This represents the space of events, marking a significant theoretical innovation in the field. Reflecting on milestones, from the Monte Bianco accident in 1999 to the present year, 2023, 24 years have passed. Similarly, two decades have passed since the introduction of Directive 2004/54. These temporal markers highlight the journey of risk assessment for tunnels and underground systems, emphasising the continuous evolution of its methodologies.

The acceptability conditions of the risk of death for tunnel users can be described through the comparative analysis of the Risk Quantum of the design exceedance probability curve and the corresponding limit of Risk Quantum in both the Acceptability and Tolerability assumed models. The availability of the Risk Quantum of all the scenarios, if properly identified, described and probabilised, allow us to calculate immediately all the requested risk indicator values. Cost-benefit versus risk analysis is also possible in the ALARP spirit.

Tunnels present a unique environment with distinct challenges due to their linear dimensions and confined conditions, which escalate the level of hazard in cases of fire, smoke, explosions, and radiation emissions, among others. The exposure and location of units made vulnerable by the various hazards align with a linear sequential modality, implying that mobility is constrained and hampered during emergency evacuations. Rescue operations are notably challenging due to these same reasons.

Italy, as a member state of the European Union, encompasses over half of the cumulative tunnel length present within the entirety of the European highway network. Given this significant tunnel infrastructure, Italy has adopted a performance-based approach for tunnel safety design, in line with the recommendations of European Directive 2004/54. This approach hinges on evaluating protection performance on one hand and quantitative risk on the other. The design process thus employs this dual methodology to ensure compliance with safety levels stipulated by regulations and decrees, in accordance with European Union recommendations.

The approach discussed in this paper stems from the Acceptability and Tolerability criteria delineated in Italian Legislative Decree No. 264/2006, pursuant to the second article of the Directive 2004/54/EC. These criteria draw on Lord Asquith’s notion, which articulates that the Risk Quantum should be “as low as reasonably practicable” for an individual. However, Italian regulations extend this notion beyond the individual risk, encompassing scenarios where multiple individuals may be exposed. Therefore, the design process must consider these collective risk scenarios. The concept of ALARP serves as the foundation for the Theory of Scenarios, where the Risk Quantum of each scenario is quantitatively measurable and the Individual Risk indicator is also measurable in the spirit of Lord Asquith judgement.

This paper presents both theoretical and practical assessments to measure scenario probabilities and design descriptions, culminating in the calculation and ranking of these design descriptions based on their Risk Quantum. The identification of the most adverse scenarios facilitates the optimisation of their design, integrating economic evaluations of different protection systems to ensure cost-effective safety solutions. Applying the Gu@lap model, once the necessary scenarios have been identified, aids in this optimisation.

The results showcased in this paper pertain to real-case design instances of a specific tunnel in Umbria, Italy. The tunnel is fully operational, featuring actual traffic, structures, and protection systems, while a parallel tunnel is currently under construction. The calculations affirm that the design complies with the law, employing a rigorous approach grounded in observed data, meticulous mathematical representation of scenarios, and thorough calculations of Risk Quantum. This dual-faceted approach, encompassing both theoretical and practical assessments, underscores the importance of a methodical and data-driven methodology in ensuring tunnel safety within the context of Italian and European regulatory frameworks.

## Figures and Tables

**Table 1:** Maximum individual risk indicator in the Tolerability and Acceptability Limit curve models that corresponds to the case of one fatality when there is one exposed unit only in the scenario.

Tunnel User	Tolerability Limit Curve	Acceptability Limit Curve
Italy (Gu)	1 × 10^−1^	1 × 10^−4^

**Table 2:** Examples of numerical limit values of Societal Risk (exceedance probability) with the Tolerability and Acceptability Limit curve models.

Fatalities	Tolerability Limit Curve (Gu) per Year	Acceptability Limit Curve (Gu) per Year
1	1 × 10^−1^	1 × 10^−4^
10	1 ×10^−2^	1 × 10^−5^
50	2 × 10^−3^	2 ×10^−6^
100	1 ×10^−3^	1 ×10^−6^

**Table 3:** Examples of numerical limit values of the Probability of Scenarios with the Tolerability and Acceptability Limit curve models (rise of the staircase-type curve of the corresponding scenario).

Fatalities	Tolerability Limit Curve (Gu) per Year	Acceptability Limit Curve (Gu) per Year
1	0.5 × 10^−1^	0.5 × 10^−4^
10	0.5 × 10^−2^	0.5 × 10^−5^

## Data Availability

The data presented in this study are available on request from the corresponding author.
